# Novel variants in the stem cell niche factor WNT2B define the disease phenotype as a congenital enteropathy with ocular dysgenesis

**DOI:** 10.1038/s41431-021-00812-1

**Published:** 2021-02-01

**Authors:** Yanjia Jason Zhang, Lissette Jimenez, Svetlana Azova, Jessica Kremen, Yee-Ming Chan, Abdelrahman M. Elhusseiny, Hajirah Saeed, Jeffrey Goldsmith, Alyaa Al-Ibraheemi, Amy E. O’Connell, Olga Kovbasnjuk, Lance Rodan, Pankaj B. Agrawal, Jay R. Thiagarajah

**Affiliations:** 1 Division of Gastroenterology, Hepatology, and Nutrition, Department of Pediatrics, Boston Children’s Hospital and Harvard Medical School, Boston, MA USA; 2 Congenital Enteropathy Program, Boston Children’s Hospital, Boston, MA USA; 3 Division of Endocrinology, Department of Pediatrics, Boston Children’s Hospital and Harvard Medical School, Boston, MA USA; 4 Department of Ophthalmology, Boston Children’s Hospital and Massachusetts Eye and Ear Infirmary, Harvard Medical School, Boston, MA USA; 5 Department of Pathology, Boston Children’s Hospital and Harvard Medical School, Boston, MA USA; 6 Division of Newborn Medicine, Department of Pediatrics, Boston Children’s Hospital and Harvard Medical School, Boston, MA USA; 7 Department of Gastroenterology, University of New Mexico School of Medicine, Albuquerque, NM USA; 8 Division of Genetics and Genomics, Department of Pediatrics, Boston Children’s Hospital and Harvard Medical School, Boston, MA USA; 9 The Manton Center for Orphan Disease Research, Boston Children’s Hospital, Boston, MA USA

**Keywords:** Disease genetics, Diarrhoea

## Abstract

WNT2B is a member of the Wnt family, a group of signal transduction proteins involved in embryologic development and stem cell renewal and maintenance. We recently reported homozygous nonsense variants in WNT2B in three individuals with severe, neonatal-onset diarrhea, and intestinal failure. Here we present a fourth case, from a separate family, with neonatal diarrhea associated with novel compound heterozygous WNT2B variants. One of the two variants was a frameshift variant (c.423del [p.Phe141fs]), while the other was a missense change (c.722 G > A [p.G241D]) that we predict through homology modeling to be deleterious, disrupting post-translational acylation. This patient presented as a neonate with severe diet-induced (osmotic) diarrhea and growth failure resulting in dependence on parenteral nutrition. Her gastrointestinal histology revealed abnormal cellular architecture particularly in the stomach and colon, including oxyntic atrophy, abnormal distribution of enteroendocrine cells, and a paucity of colonic crypt glands. In addition to her gastrointestinal findings, she had bilateral corneal clouding and atypical genital development later identified as a testicular 46,XX difference/disorder of sexual development. Upon review of the previously reported cases, two others also had anterior segment ocular anomalies though none had atypical genital development. This growing case series suggests that variants in WNT2B are associated with an oculo-intestinal (and possibly gonadal) syndrome, due to the protein’s putative involvement in multiple developmental and stem cell maintenance pathways.

## Introduction

The Wnt family of signal transduction proteins comprises a diverse family of secreted lipid-modified signaling glycoproteins that are involved in embryologic development and in maintenance and renewal of pluripotent stem cells throughout the body. In the intestine Wnt proteins are well-described to be critical in the homeostasis of multiple cellular compartments including the rapidly renewing intestinal epithelium that mediates nutrient and fluid transport between the environment and the body [1–3]. In mouse models, multiple Wnts including WNT2B are expressed in the intestine and mesenchymal stromal cells have been identified as a key source of secreted Wnt proteins [4]. All Wnt proteins are obligately palmitoylated by Porcupine (Porcn), which allows Wnt secretion and binding to Frizzled receptors [5]. Loss of Wnt signaling has been shown to negatively affect intestinal stem cell (ISC) maintenance and proliferation, resulting in epithelial disruption, crypt drop out, and enteroid failure [1, 6, 7]. Thus, mesenchymal Wnt secretion is essential for maintaining the ISC niche and intestinal epithelial homeostasis.

In humans, WNT2B is a Wnt family member that has recently been associated with a group of intestinal disorders termed Congenital Diarrheas and Enteropathies (CODEs) [8]. CODEs are a heterogeneous group of genetic disorders characterized by severe neonatal-onset diarrhea that often require significant nutritional and fluid support [8]. While many categories of pathology can lead to CODEs, several CODE-associated genes are involved in maintaining intestinal epithelial architecture. Unique homozygous nonsense variants in WNT2B were first associated with CODE in three patients from two families who had neonatal-onset diarrhea, growth failure, and years-long parenteral nutrition (PN) dependence [3]. They had architectural distortion throughout the gastrointestinal mucosa and their clinical manifestations were most consistent in the gastrointestinal tract. In this report we identify a patient with a similar clinical phenotype who was subsequently found to have novel compound heterozygous variants in *WNT2B*. Both variants are novel. One is a missense variant and the other is a frameshift variant; both are predicted to be deleterious. We show through in silico structural modeling that the missense variant occurs near the conserved serine that is the target for acylation, and that the substitution likely prevents the essential acylation, and thereby disrupts subsequent downstream Wnt signaling.

Interestingly, our patient also had prominent extra-intestinal manifestations that may be related to her *WNT2B* variants. These included microcorneas and corneal clouding as well as development of testes in the context of an XX sex-chromosome complement. Two other patients seen in our institution with *WNT2B* variants had ocular findings as well, including microcorneas and colobomas extending from the iris to the retina. In summary, we present a case with novel WNT2B variants associated with congenital diarrhea that confirms the gastrointestinal phenotype associated with loss of WNT2B function, suggests that ocular involvement is a central feature of the syndrome and that WNT2B palmitoylation is important in human intestinal homeostasis.

## Materials (subjects) and methods

### Whole-exome and Sanger sequencing (WES)

DNA from proband I-4 and mother were sent to GeneDx for whole-exome sequencing (WES) [9]. A sequencing library was prepared using the Illumina Exome Enrichment Protocol and captured libraries were sequenced on Illumina HiSeq 2000 or 4000. Sequences were aligned to the human genome reference sequence (hg19). The sequences were aligned to the reference sequence using BWA (Burrows–Wheeler Aligner, version 0.7.15) and variants called with Gatk best practices (version 3.7). The data were filtered to include variants with an allele frequency of <0.001 in publicly available normative databases (gnomAD). Variants were identified using the WuXi NextCode platform. Missense variants were examined using multiple techniques. Amino acid conservation was determined by comparing the human sequence with eight species (chimpanzee, rat, mouse, dog, chicken, zebrafish, fruit fly, and *Caenorhabditis* *elegans*). Physiochemical differences between the canonical and patient amino acid sequences were determined using CADD, and PolyPhen-2, MutationTaster and SIFT were also used to estimate the impact of the variant on DNA and protein.

Segregation of the identified *WNT2B* variants in patient I-4 was confirmed by Sanger sequencing for available family members. The relevant portion of the gene was PCR amplified and Sanger sequencing was performed. The bi-directional sequence was assembled, aligned to reference gene sequences based on human genome build GRCh37/UCSC hg19 and analyzed for known familial sequence variant(s). Sequence alterations were reported according to the Human Genome Variation Society (HGVS) nomenclature guidelines. The variants reported here are deposited in ClinVar (National Center for Biotechnology Information. ClinVar; WNT2B c.423delTp.F141LfsX10: VCV000984423.1; WNT2B c.722 G > A p.G241D: VCV000984424.1

### Histology

Intestinal and gonadal biopsy samples from affected individuals, were paraffin-embedded and sectioned. Staining was done with hematoxylin & eosin (H&E) per standard clinical protocol.

### Homology modeling and visualization of WNT2B structure

The amino acid sequences of human WNT3 (Gene ID 7473) and WNT2B (Gene ID 7482) were retrieved from the GenBank database. The latter was used as the target for protein structure modeling using the SWISS-MODEL server. The server searched existing X-ray structures in PDB for best templates. The WNT3 structure (PDB: 6AHY), which we expected to be the closest available template, also had the best Global Model Quality Estimation and QMEAN statistical parameters and was thus chosen as a template. We imported the homology model of WNT2B to PyMol Molecular Graphics System, Version 2.3.3, Schrödinger, LLC for further visualization, highlighting variants, and producing images.

### RNAscope

Formalin-fixed tissue sections on glass slides were deparaffinized in xylene following a gradient of ethanol solutions and prepared for a staining with RNAscope Multiplex Fluorescent V2 using either human *WNT2B* or negative or positive control probes. Sample preparation and labeling were performed according to the manufacturer’s protocol (ACDBio, Newark, CA). Opal fluorophores (Perkin Elmer, Waltham, MA) and DAPI were used for visualization. 50 μL of FluorSave Reagent was added to the tissue samples, which were mounted between a glass slide and the coverslip. Confocal imaging was carried out in using a Zeiss LSM 800 AiryScan confocal microscope using Zen software.

## Results

### Identification of novel *WNT2B* variants

**Fig. 1:**
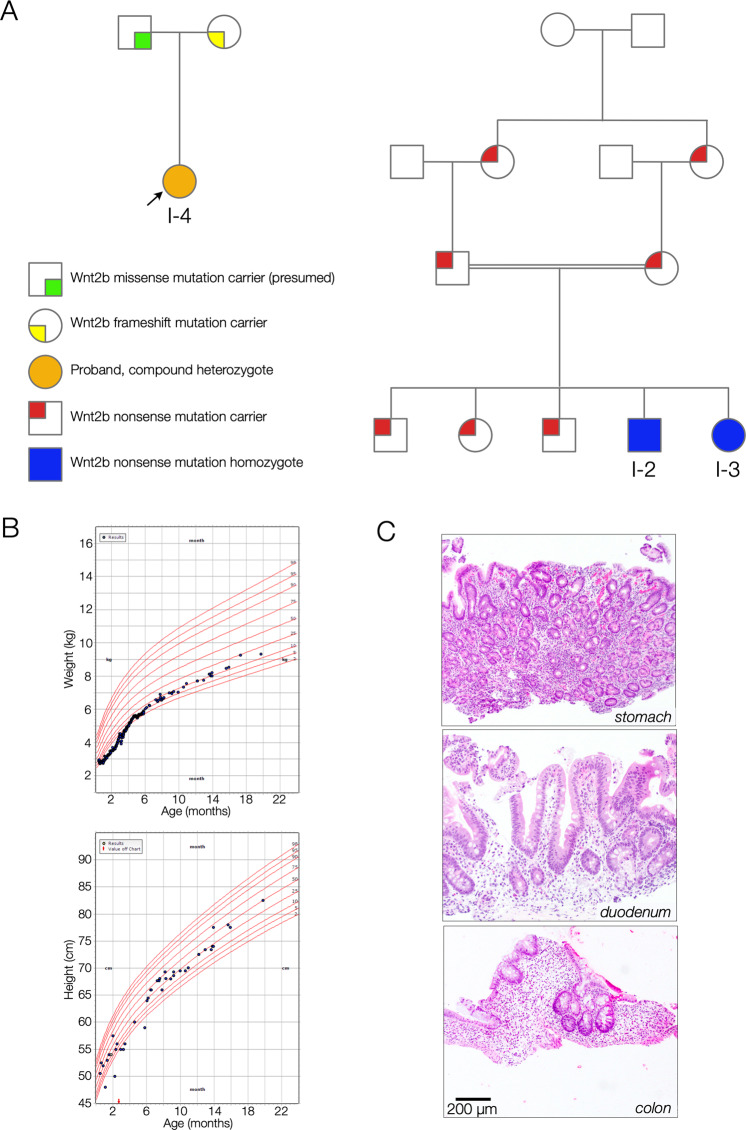
Pedigree, growth charts, and intestinal histology. **A** Pedigrees for I-4 (left) and I-2 and I-3 (right). **B** Growth charts showing weight (top) and height (bottom) for patient I-4. **C** H&E-stained tissue sections obtained from endoscopic biopsies from the body of the stomach, proximal duodenum, and right colon for patient I-4. The stomach shows full-thickness lamina propria chronic inflammation and partial oxyntic atrophy. The duodenum shows equivocally increased lamina propria chronic inflammation, and the colon shows conspicuous crypt dropout with increased lamina propria lymphoplasmacytic inflammation.

A female patient (I-4) of Haitian descent with neonatal-onset diarrhea, corneal clouding and atypical genital appearance was referred to our institution (Boston Children’s Hospital) for multidisciplinary care. WES revealed two novel variants in the *WNT2B* gene. Notably, the three previously reported patients (I-1, I-2, and I-3) with *WNT2B* variants and associated congenital diarrhea all had homozygous nonsense variants (c.205 C > T [p.Arg69*] and c.313 C > T [p.Arg105*]) (Fig. 1A) [3]. Our patient’s sequencing revealed novel coding variants in *WNT2B (NM_024494.3)*. She and her mother share the c.423del [p.Phe141fs] variant, which is predicted to be a deleterious frameshift variant. Additionally, our patient carries a missense variant, c.722 G > A [p.G241D], which presumably arose de novo or was inherited from her father, who was not enrolled in the study (Fig. 1A).

### Gastrointestinal clinical presentation and nutritional features

Our patient (I-4) was observed to have neonatal onset diarrhea. At 2 weeks of age, she presented with metabolic acidosis (serum bicarbonate 9 mmol/L) and failure to thrive in the setting of progressively diffuse watery diarrhea that occurred up to ten times per day since birth. An initial elemental formula trial did not alter diarrheal frequency, with symptoms improving only after enteral nutrition was withheld and the patient was initiated on PN. At 1 month of age, endoscopic examination was performed and visually notable for decreased ruggae, with biopsies revealing gastric oxyntic atrophy with chronic inflammation, mild chronic inflammation in the duodenum, and crypt dropout in the colon (Fig. 1C). Given these findings, she was initially treated for presumed autoimmune enteropathy with a course of methylprednisolone and sirolimus (goal trough 5–10 ng/mL) with an inconsistent clinical response in stool output and PN support. At 5 months of age, repeat endoscopy showed the same mucosal architectural alterations, at which point immune-targeted therapies were stopped.

Over the course of the patient’s hospitalization, enteral advancement via a nasogastric tube was trialed and between 3 and 4 months of age she was able to tolerate a slow enteral advancement of an elemental formula. She was able to briefly wean off PN around 4 months of age but was subsequently restarted due to concern for worsening dehydration and hypernatremia in the setting of infection. She continues to remain largely dependent on PN support with enteral advancement hindered by oral aversion. Gastrointestinal symptoms are minimal in the setting of PN support, with soft, non-bloody bowel movements 2–3 times per day and without vomiting or abdominal pain.

Her parenteral fluid support requirements have remained stable over time approximating average-for-weight maintenance fluid needs. Her laboratory studies have consistently demonstrated a mild metabolic acidosis with serum bicarbonate levels ranging between 17 and 23 mmol/L (reference [ref] range 17–29 mmol/L) and requiring at least ¾ acetate supplementation in her PN. Micronutrient review has been notable for iron deficiency anemia with the patient absorbing and tolerating enteral iron supplementation. She is currently achieving age- and sex-appropriate growth, while on PN (Fig. 1B).

### Ophthalmologic features

**Fig. 2:**
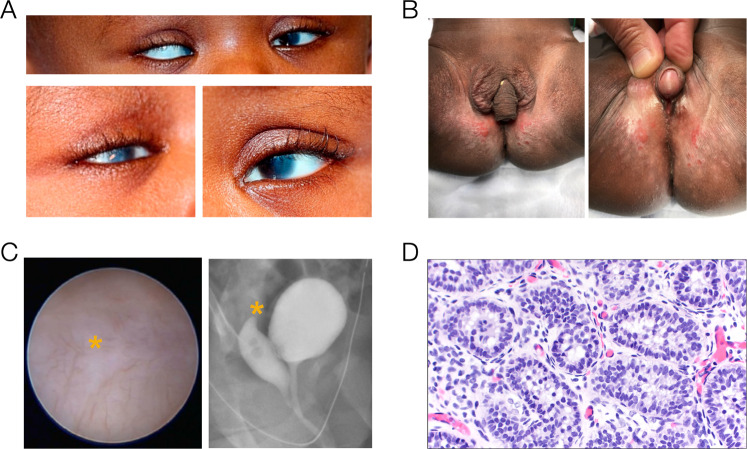
Ophthalmologic and gonadal features. **A** Top panel: Photo of the patient’s eyes as she is gazing at the viewer. Note the large esotropia of the right eye. Bottom left panel: Corneal clouding of the right eye that covers the visual axis and extends to the limbus. Bottom right panel: Corneal clouding of the left eye extends partially into the visual axis. **B** Ambiguous external genitalia in patient I-4. **C** Cystoscopy (left) and fluoroscopic genitogram (right) showing absent cervical os (asterisks). **D** H&E-stained gonadal tissue section showing testicular parenchyma containing primitive seminiferous tubules. No ovarian tissue is identified.

At birth, ocular evaluation of our proband (I-4) revealed bilateral microcornea and corneal clouding, and a right large angle sensory esotropia of >50 prism diopters. The opacity of the right cornea involves the central cornea and extends peripherally to the limbus while the opacity in the left cornea is peripheral and extends partially across the visual axis (Fig. 2A). Upon further review of other patients with *WNT2B* variants, we noted that two other patients under our institution’s care also had anterior segment anomalies, as detailed in our case comparison below.

### Endocrine manifestations

The patient’s mother initially received prenatal care in Haiti, where several early ultrasounds documented typical female-appearing external genitalia. Upon arrival to the United States, a third trimester ultrasound identified atypical genital appearance. A uterus could not be visualized. No further diagnostic genetic evaluation or imaging was performed. Postnatal examination showed the presence of a clitorophallic structure, measuring 2.2 cm in length and 1 cm in diameter, with a ventral curvature, a hypospadic meatus at the ventral base, and mild penoscrotal transposition (Fig. 2B). There was posterior labioscrotal fusion, with an anogenital ratio of 0.76 (typical female <0.5). Labioscrotal folds were hyperpigmented with rugae. A gonad was palpable in the right inguinal canal; the left gonad was not palpable. Karyotype was 46,XX and fluorescence in situ hybridization analysis did not identify an *SRY* sequence. The newborn screen showed normal 17-hydroxyprogesterone and a biochemical congenital adrenal hyperplasia panel did not suggest any enzymatic defects in adrenal steroid biosynthesis. Hormonal studies at 2 weeks of life showed LH 11.76 IU/L (ref range 0.02–7.0 IU/L), FSH 5.24 IU/L (ref range for female infants 1.2–12.5 IU/L), estradiol <20.0 pg/mL, testosterone 137 ng/dL (ref range for females <1 month 20–64 ng/mL), and anti-Müllerian hormone (AMH) 60.175 ng/mL (ref range for females 0.53–7.78 ng/mL). Abdominal/pelvic ultrasound confirmed the presence of a right inguinal gonad that was solid in appearance and identified a left intraabdominal gonad with a solid component and an adjacent multicystic structure. A blind-ending fluid-filled structure was demonstrated in the midline pelvis between the bladder and rectum without a cervical impression, but no normal-appearing uterus was visualized.

A genetic panel for differences/disorders of sex development (DSD) did not identify any variants in genes previously implicated in *SRY*-negative XX testicular DSD, including *NR5A1*, *SOX9*, *SOX3*, *WNT4*, *RSPO1*, and *WT1*. The patient also had cystoscopy, vaginoscopy, and laparoscopy, which demonstrated a urogenital sinus and absence of the cervix and uterus (Fig. 2C). Bilateral gonadal biopsy performed at 9 months of age demonstrated testicular parenchyma with primitive seminiferous tubules and no evidence of ovarian tissue (Fig. 2D). These findings were consistent with a diagnosis of 46,XX testicular DSD leading to production of testosterone, which caused virilization of the external genitalia, and AMH, which caused the absence of the uterus.

### Intestinal expression of WNT2B

**Fig. 3:**
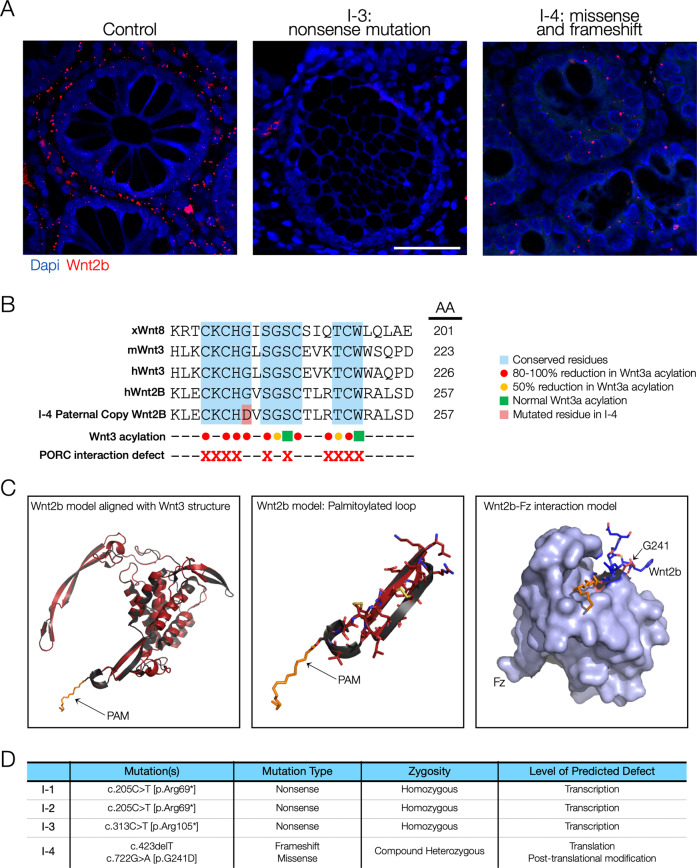
Wnt2b tissue expression and missense variant structural analysis. **A** In situ hybridization of Wnt2b mRNA (RNAscope) showing Wnt2B expression (red dots) in control (left panel), patient I-3 (middle panel) and patient I-4 (right panel) in colon biopsies. RNA expression is abolished in I-3 but intermediate in I-4. **B** Alignment of Wnt homologs. xWnt8: Xenopus Wnt8; mWnt3: Murine Wnt3; hWnt3: Human Wnt3; hWnt2B: Human Wnt2B. Patient I-4 G241D variant is highlighted in red. Residues of mWnt3 known to be essential for acylation are noted with red dots, nonessential for acylation are noted with green squares. Red Xs denote mWnt3 residues required for interaction with Porcupine, the mWnt3 acyltransferase. G241, the mutated residue in patient I-4, is predicted to negatively affect palmitoylation. **C** Homology modeling of hWnt2B based on hWnt3 structure. Left panel: hWnt2B model (red) aligned with hWnt3 structure (black) with palmitoyl group (PAM, orange) noted. Middle panel: same alignment zoomed in on palmitoylated loop. Right panel: hWnt2B model (blue sticks) with PAM moiety (orange) interaction with Frizzled (Fz, blue-gray). G241, the mutated residue in patient I-4, sits above the binding pocket and is not predicted to affect binding. **D** Table of variants of the four individuals in our case series and predicted synthetic defect.

We sought to identify the mechanism by which the *WNT2B* variants in our series abrogate WNT2B protein function. We first measured mRNA expression in gastrointestinal biopsy tissue, hypothesizing that nonsense variants would result in loss of expression through nonsense-mediated decay, but that missense variants may permit some level of mRNA expression. Fluorescent RNA hybridization and confocal imaging supported this prediction. Colonic sections from control patients showed widespread expression of *WNT2B* mRNA in both the lamina propria and stromal cells as well as colonic crypts. In contrast, in tissue obtained from patient I-3 (homozygous nonsense variants) there was no appreciable detection of *WNT2B* mRNA. Interestingly, we did find expression, albeit significantly decreased in comparison to control tissue, in our novel patient I-4 (compound heterozygous variants with one missense allele) (Fig. 3A).

### In silico structural modeling

We next sought to understand how the G241D substitution results in a nonfunctioning protein. To that end, we created a structural model of human WNT2B using Swiss Model and the recently cataloged structure of WNT3 [10]. This model positioned Gly241 in a highly conserved hairpin loop near Ser243, which was predicted to be a palmitoylation site. S-palmitoylation is a conserved post-translational modification necessary for binding to Frizzled receptors, the canonical receptor for Wnt proteins in the Wnt/beta-catenin signaling pathway. Mutagenesis studies of the murine Wnt3 homolog previously demonstrated that the conserved glycine residue is essential for interaction with the acyltransferase Porcn and therefore necessary for S-palmitoylation (Fig. 3B) [5, 11]. We further modeled the WNT2B-Frizzled interaction using the WNT3B-Frizzled complex structure, which suggests a key role of the palmitoyl group for interaction with the substituted glycine reside sitting above the interaction groove (Fig. 3C). Altogether, we show that nonsense variants (represented by I-2 and I-3) result in tissue-level transcription defects and predict that the missense variant (I-4) results in normal transcription and translation but an inability to receive its key post-translational modification (Fig. 3D).

### Case comparisons: an oculo-intestinal syndrome

**Fig. 4:**
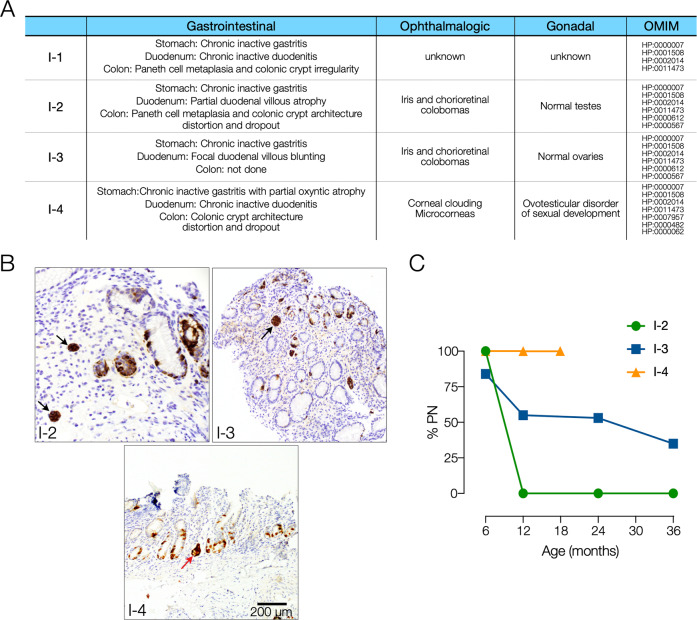
Wnt2b syndrome features and nutritional outcomes. **A** Shared clinical features of patients with WNT2B variants. **B** Stomach (antrum) tissue sections stained for chromogranin (brown) showing linear (red arrow) and micronodular (black arrow) enterochromaffin-like cell hyperplasia in patients I-2, I-3, and I-4. **C** Parenteral nutrition requirements for adequate growth in patients I-2, I-3, and I-4. Age, weight, height and school level at last evaluation: I-2—6 years-old, 16 kg (*z*-score −2.92) 107 cm (*z*-score −2.55), elementary; I-3—3 years-old, 12.7 kg (*z*-score −1.10), 87 cm (*z*-score −2.26), pre-school; I-4—18 months-old, 9.32 kg (*z*-score −1.06), 82.5 cm (*z*-score 0.00), pre-school.

All our patients had either predicted or demonstrated loss of WNT2B signaling, either at the level of transcription or at the level of post-translational acylation and secretion. We performed a clinical comparison to determine the syndromic effect of WNT2B signaling loss (Fig. 4A). All four patients had GI pathology characterized by abnormal intestinal architecture particularly notable in the stomach and the colon along with mild nonspecific chronic inflammation. A notable pathologic feature shared between all our patients was the presence of striking enteroendocrine cell hyperplasia in the absence of significant inflammation (Fig. 4B). All four patients required PN early in life, though all tolerated at least some enteral advancement over time, including one patient who completely weaned off of PN at one year of age (Fig. 4C). Our patients had most success advancing enteral nutrition when advanced as continuous feeds via a gastrostomy tube with a cow’s milk-based formula. All individuals had limited oral intake, though none had need for dietary restrictions. Patient I-3 required additional enteral bicarbonate supplementation with enteral advancement. Of interest, two patients were noted to have alterations in their enteral tolerance after a systemic illness. Worsening stooling frequency with enteral advancement, electrolyte derangements, and emesis were the common barriers for decreasing PN support. The three patients in our institution’s series had ocular findings. In addition to the patient detailed above, patient I-2 had bilateral microcornea, corneal neovascularization, and thick corneas, while patient I-3 had bilateral iridocorneal adhesions, congenital cataract, and iris coloboma. Interestingly, patient I-4 uniquely had gonadal findings, while the other three had no gonadal or endocrinologic defects.

## Discussion

Here, through clinical, molecular and in silico investigation of a series of patients with a diversity of *WNT2B* variants, we show the importance of WNT2B in the development of multiple organ systems. We demonstrate that human *WNT2B* variants result in an oculo-intestinal syndrome with specific gastrointestinal pathology. We further describe a novel WNT2B variant that suggests the importance of palmitoylation for signaling, consistent with the known biology of Wnt proteins. Finally, through long-term follow up of the known patients with this rare disorder, we show a favorable nutritional prognosis over time and the ability for at least partial weaning of PN.

From a gastroenterological perspective, all patients with *WNT2B* variants presented with neonatal-onset diarrhea and chronic inflammatory changes throughout their gastrointestinal tract [3]. The other pertinent shared histologic feature is abnormal intestinal cellular architecture throughout the gastrointestinal tract. The most striking histological changes are seen in the stomach and colon in all patients, which may suggest that WNT2B is particularly critical for development and mucosal homeostasis in these gastrointestinal regions. All patients had some variable degree of chronic mucosal inflammation, although this may be due to abnormal intestinal development and loss of barrier function rather than an intrinsic underlying immune-dysregulation. Our patient’s histological findings included a persistent paucity of colonic crypts, suggesting reduced regenerative capacity and consistent with a loss of production of new epithelial cells. In the stomach this was accompanied by enteroendocrine cell hyperplasia which can be seen during generalized chronic inflammation or autoimmune gastritis. However, our patients had highly increased chromogranin staining with either mild or absent inflammation suggesting a noninflammatory mechanism for enteroendocrine cell hyperplasia. Along with altered regenerative capacity, this may suggest a role for WNT2B in intestinal epithelial progenitor cell differentiation and specification. This may explain our observation of reduced enteral nutrition tolerance after systemic infection. Consistent with this speculation, our in silico modeling predicts an absence of WNT2B secretion and binding to Frizzled receptors, which is known to be important for the maintenance of ISCs and progenitor cell populations [6]. A lack of WNT2B signaling may therefore lead to an imbalance of stem cell fate signals leading to premature differentiation and hyperplasia of certain cell types. Finally, in keeping with the concept that the inflammatory response is secondary, treatment with steroids and sirolimus was ineffective for the patient described in this case.

The multisystem involvement in our patient suggests that perturbations in WNT2B signaling may have widespread effects that could involve gonadal development. Previous studies have established the role of WNT4 and R-SPONDIN-1 (RSPO1) in promoting ovarian differentiation of the gonadal primordium via activation the Wnt/β-catenin signaling pathway, possibly through inhibition of genes involved in testicular fate determination. Accordingly, loss of function variants in *WNT4* and *RSPO1* have been linked to virilizing testicular/ovotesticular DSDs in 46,XX, SRY-negative individuals, while gain-of-function variants in genes that activate the β-catenin pathway have been associated with 46,XY DSD [12–17].

Recent work has also implicated WNT2B in ovarian development with WNT2B expression upregulated in human fetal ovary during a critical period of differentiation [18]. Localization of WNT2B to ovarian surface epithelial cells between days 45 and 73 of gestation suggests a possible role in the survival and migration of pre-granulosa cells to the epithelial layer [18, 19]. This could be analogous to the inability of WNT2B-deficient intestinal cells to generate organoids in vitro. Our patient’s genetic testing confirmed a maternally inherited frameshift variant in WNT2B. The second missense variant in WNT2B, in trans to the frameshift variant, was either paternally inherited (father was unavailable for genetic testing) or was de novo. The mother underwent ovulation induction prior to this pregnancy but denied any history of virilization. The three probands previously described with WNT2B-associated congenital diarrhea had no gonadal phenotype. However, all of these individuals had homozygous nonsense WNT2B variants, presumably leading to a lack of functioning protein product [3]. It is possible that the unique gonadal findings in the patient described in this report were a consequence of the accumulation of an abnormal un-palmitoylated protein product that interfered with Wnt/β-catenin signaling by exerting a dominant negative effect. Similar gain of function effects in non-intestinal sites have been recently described in patients with congenital diarrhea [20]. Given the known roles of WNT4 and R-SPONDIN-1 in ovarian development, future functional studies may be performed to test the validity of this hypothesis and to define the role of WNT2B in gonadal development and differentiation. Alternatively, our proband may carry a yet unidentified gene variant that may explain the gonadal phenotype.

All three patients in our institution with *WNT2B* variants had ophthalmologic findings. *WNT2B* has been implicated in retinal development and stem cell maintenance in multiple model organisms. Fokina et al. demonstrated in chicken models that the *WNT2B* gene and other *WNT* genes (*WNT6* and *WNT9B*) are expressed in the ocular surface ectoderm including corneal epithelium, anterior lens epithelium, and iris [21]. Interestingly, *WNT2B* expression in the developing chick eye localizes to the ciliary margin, which, similar to intestinal crypts, is a stem cell reservoir. Wnt signaling pathways are involved in lens and ocular ectoderm development and are hypothesized to regulate corneal cellular proliferation [21]. Variants of these genes may affect the development of the anterior segment and result in corneal clouding, iridocorneal adhesions, congenital cataracts, and iris colobomas.

We also present tissue-level expression of WNT2B for the patients in our series along with in silico modeling of WNT2B. The previously described nonsense variants and the novel frameshift variant are predicted to result in complete loss of protein activity [3]. However, it was less clear how the G241D substitution results in aberrant protein function. Our model shows that (1) the glycine residue mutated in our case is conserved across species and across the family of WNT proteins, (2) mutation of this conserved glycine in murine WNT3 is predicted to result in a loss of nearby S-palmitoylation and (3) the S-palmitoyl structure (and not the mutated glycine itself) is likely important for binding to a Frizzled receptor [5, 10, 11]. Based on this structural evidence, we therefore propose that the G241D variant results in defective post-translational acylation of WNT2B. Further functional studies are needed to test this prediction by measuring relative protein expression (which we hypothesize to be normal) and acyl-serine’s (which we predict to be decreased) through tandem mass spectroscopy in control and patient tissues.

In summary, we add to a growing case series by describing the fourth known patient with congenital diarrhea and novel deleterious variants in *WNT2B*. Structural modeling suggests that the novel missense variant prevents post-translational acylation, which subsequently abrogates binding to the Frizzled receptor. The case we present has prominent extra-intestinal features, with ophthalmologic findings likely associated with *WNT2B* variants. Pre-clinical data in animal models (for the eye) and ex-vivo human tissue (for the gonads) point to a role of *WNT2B* in organ-specific stem cell maintenance [18, 21, 22]. A similar role could explain the abnormalities in cellular architecture in our patient’s gastrointestinal histology and could be the reason why *WNT2B*-deficient cells are unable to form intestinal organoids [3]. Together, this expanded case series demonstrates that loss of WNT2B signaling results in an oculo-intestinal syndrome with unique intestinal histological findings and a possible gonadal phenotype consistent with the involvement of WNT2B in multiple developmental pathways.

## References

[1] Farin HF, Es JHV, Clevers H (2012). Redundant sources of Wnt regulate intestinal stem cells and promote formation of paneth cells. Gastroenterology.

[2] Ortiz-Masià D, Salvador P, Macias-Ceja DC, Gisbert-Ferrándiz L, Esplugues JV, Manyé J (2019). WNT2b activates epithelial-mesenchymal transition through FZD4: relevance in penetrating Crohn´s disease. J Crohn’s Colitis.

[3] O’Connell AE, Zhou F, Shah MS, Murphy Q, Rickner H, Kelsen J (2018). Neonatal-onset chronic diarrhea caused by homozygous nonsense WNT2B mutations. Am J Hum Genet.

[4] Shoshkes-Carmel M, Wang YJ, Wangensteen KJ, Tóth B, Kondo A, Massasa EE (2018). Subepithelial telocytes are an important source of Wnts that supports intestinal crypts. Nature.

[5] Nile AH, Hannoush RN (2016). Fatty acylation of Wnt proteins. Nat Chem Biol.

[6] Santos AJM, Lo Y-H, Mah AT, Kuo CJ (2018). The intestinal stem cell niche: homeostasis and adaptations. Trends Cell Biol.

[7] Es JH, van, Haegebarth A, Kujala P, Itzkovitz S, Koo B-K, Boj SF (2012). A critical role for the Wnt effector Tcf4 in adult intestinal homeostatic self-renewal. Mol Cell Biol.

[8] Thiagarajah JR, Kamin DS, Acra S, Goldsmith JD, Roland JT, Lencer WI (2018). Advances in evaluation of chronic diarrhea in infants. Gastroenterology.

[9] Rockowitz S, LeCompte N, Carmack M, Quitadamo A, Wang L, Park M (2020). Children’s rare disease cohorts: an integrative research and clinical genomics initiative. Npj Genom Med.

[10] Hirai H, Matoba K, Mihara E, Arimori T, Takagi J (2019). Crystal structure of a mammalian Wnt–frizzled complex. Nat Struct Mol Biol.

[11] Rios-Esteves J, Haugen B, Resh MD (2014). Identification of key residues and regions important for porcupine-mediated Wnt acylation. J Biol Chem.

[12] Biason-Lauber A, Konrad D, Navratil F, Schoenle EJ (2004). AWNT4mutation associated with Müllerian-duct regression and virilization in a 46,XX woman. N. Engl J Med.

[13] Mandel H, Shemer R, Borochowitz ZU, Okopnik M, Knopf C, Indelman M (2008). SERKAL syndrome: an autosomal-recessive disorder caused by a loss-of-function mutation in WNT4. Am J Hum Genet.

[14] Chassot A-A, Gillot I, Chaboissier M-C (2014). R-spondin1, WNT4, and the CTNNB1 signaling pathway: strict control over ovarian differentiation. Reprod Camb Engl.

[15] Maatouk DM, DiNapoli L, Alvers A, Parker KL, Taketo MM, Capel B (2008). Stabilization of beta-catenin in XY gonads causes male-to-female sex-reversal. Hum Mol Genet.

[16] Granados A, Alaniz VI, Mohnach L, Barseghyan H, Vilain E, Ostrer H (2017). MAP3K1-related gonadal dysgenesis: six new cases and review of the literature. Am J Med Genet Part C Semin Med Genet.

[17] Loke J, Pearlman A, Radi O, Zuffardi O, Giussani U, Pallotta R (2013). Mutations in MAP3K1 tilt the balance from SOX9/FGF9 to WNT/β-catenin signaling. Hum Mol Genet.

[18] Mamsen LS, Ernst EH, Borup R, Larsen A, Olesen RH, Ernst E (2017). Temporal expression pattern of genes during the period of sex differentiation in human embryonic gonads. Sci Rep.

[19] Ricken A, Lochhead P, Kontogiannea M, Farookhi (2002). Watermark-silverchair-com.ezp-prod1.hul.harvard.edu 1/24/2020, 5:08:33 PM.pdf. Endocrinology.

[20] Overeem AW, Li Q, Qiu Y, Cartón‐García F, Leng C, Klappe K (2020). A molecular mechanism underlying genotype‐specific intrahepatic cholestasis resulting from MYO5B mutations. Hepatology.

[21] Fokina VM, Frolova EI (2006). Expression patterns of Wnt genes during development of an anterior part of the chicken eye. Dev Dynam.

[22] Ohta K, Ito A, Kuriyama S, Lupo G, Kosaka M, Ohnuma S-i (2011). Tsukushi functions as a Wnt signaling inhibitor by competing with Wnt2b for binding to transmembrane protein Frizzled4. Proc Natl Acad Sci.

